# Application of 233 nm far-UVC LEDs for eradication of MRSA and MSSA and risk assessment on skin models

**DOI:** 10.1038/s41598-022-06397-z

**Published:** 2022-02-16

**Authors:** Paula Zwicker, Johannes Schleusener, Silke B. Lohan, Loris Busch, Claudia Sicher, Sven Einfeldt, Michael Kneissl, Anja A. Kühl, Cornelia M. Keck, Christian Witzel, Axel Kramer, Martina C. Meinke

**Affiliations:** 1 Institute of Hygiene and Environmental Medicine, University Medicine Greifswald, Ferdinand-Sauerbruch-Str., 17475 Greifswald, Germany; 2 Center of Experimental and Applied Cutaneous Physiology, Department of Dermatology, Venerology and Allergology, Charité-Universitätsmedizin Berlin, Corporate Member of Freie Universität Berlin and Humboldt-Universität zu Berlin, Charitéplatz 1, 10117 Berlin, Germany; 3 Department of Pharmaceutics and Biopharmaceutics, Philipps-Universität Marburg, Robert-Koch-Str. 4, 35032 Marburg, Germany; 4 Ferdinand-Braun-Institut gGmbH, Leibniz-Institut Für Höchstfrequenztechnik, Gustav-Kirchhoff-Str. 4, 12489 Berlin, Germany; 5 Institute of Solid State Physics, Technische Universität Berlin, Hardenbergstr. 36, 10623 Berlin, Germany; 6 iPATH.Berlin-Immunopathology for Experimental Models, Core Facility of the Charité-Universitätsmedizin Berlin, Corporate Member of Freie Universität Berlin and Humboldt-Universität zu Berlin, Charitéplatz 1, 10117 Berlin, Germany; 7 Division of Plastic and Reconstructive Surgery, Department of Surgery, Charité-Universitätsmedizin Berlin, Corporate Member of Freie Universität Berlin and Humboldt-Universität zu Berlin, Charitéplatz 1, 10117 Berlin, Germany

**Keywords:** Risk factors, Skin cancer, DNA

## Abstract

A newly developed UVC LED source with an emission wavelength of 233 nm was proved on bactericidal efficacy and skin tolerability. The bactericidal efficacy was qualitatively analysed using blood agar test. Subsequently, quantitative analyses were performed on germ carrier tests using the MRSA strain DSM11822, the MSSA strain DSM799, *S. epidermidis* DSM1798 with various soil loads. Additionally, the compatibility of the germicidal radiation doses on excised human skin and reconstructed human epidermis was proved. Cell viability, DNA damage and production of radicals were assessed in comparison to typical UVC radiation from discharge lamps (222 nm, 254 nm) and UVB (280–380 nm) radiation for clinical assessment. At a dose of 40 mJ/cm^2^, the 233 nm light source reduced the viable microorganisms by a log_10_ reduction (LR) of 5 log_10_ levels if no soil load was present. Mucin and protein containing soil loads diminished the effect to an LR of 1.5–3.3. A salt solution representing artificial sweat (pH 8.4) had only minor effects on the reduction. The viability of the skin models was not reduced and the DNA damage was far below the damage evoked by 0.1 UVB minimal erythema dose, which can be regarded as safe. Furthermore, the induced damage vanished after 24 h. Irradiation on four consecutive days also did not evoke DNA damage. The radical formation was far lower than 20 min outdoor visible light would cause, which is classified as low radical load and can be compensated by the antioxidant defence system.

## Introduction

Methicillin-resistant *Staphylococcus aureus* (MRSA) belong to the most common multi-resistant pathogens. The primary location of MRSA, as well as Methicillin-sensitive *Staphylococcus aureus* (MSSA), is the nasal vestibule that is considered as an initial point for colonization of the body. Both are the main origin of surgical site infections (SSI). If an MRSA carrier is identified preoperatively, an indication for decolonization is given, since the increased risk for SSI resulting in prolonged hospital stays and increased mortality will be reduced^1,2^. MSSA are an independent risk factor for the colonization of alloplastic implants; nowadays the universal decolonization of MSSA is increasingly gaining importance^3^.

The decolonization with the conventional antibiotic Mupirocin leads to resistances^4^, followed by decreased efficacy^5^. As an alternative, nasal decolonization is performed with antiseptic agents, such as chlorhexidine digluconate or octenidine dihydrochloride, which are either insufficient^6^ or result in formation of resistances with cross-resistance to antibiotics, questioning its use^7^. Thus, a new microbiocidal surgical method that does not evoke formation of resistances is desirable. Therefore, the application of UVC radiation is a suitable and promising alternative^8^.

Previous work has shown that MRSA can be eradicated with UVC radiation at a wavelength of 254 nm and more recently also at 222 nm^9,10^.

UV radiation is absorbed by proteins and nucleic acids as DNA, promoting the development of molecular rearrangements and photoproducts, such as the major DNA damage products cyclobutane pyrimidine dimers (CPD) and pyrimidine(6-4) pyrimidone photoproducts (6-4PPs), which are associated with the development of mutations and cancer^11,12^.

After irradiation with 254 nm, about 70% of the basal cells in the skin are negatively affected in their vitality^13^, while almost no pre-mutagenous UV-associated DNA lesions were observed with 222 nm, which can be explained by the higher absorption of 254 nm wavelength by proteins and nucleic acids—being associated with the development of mutations leading to skin cancer^11,12^ and eye cataracts^14,15^. However, high energy UVC radiation (*λ* < 245 nm) is strongly absorbed by the stratum corneum (SC), the horny layer that does not contain cell nuclei^16^. In contrast, small microbes (< 1 µm) can be efficiently inactivated on the surface of the skin^17^ as already shown on MRSA that could be eradicated by using 222 nm radiation on 3D murine skin^16^.

Another key aspect is radical formation which is mainly induced by UVA but also by UVB, visible, and in low amounts also near infrared light^18^. In order to prevent enhanced tumour development^19,20^ as a consequence of cell and tissue damage^21–23^ by increased oxidation of different cell components, radicals need to be tightly controlled. So far, effects on the radical production in skin due to UVC irradiation have not been published.

In this study, the inactivation of different bacterial strains as well as the effect on skin using a recently developed 233 nm far-UVC LED source^24,25^ were evaluated and compared to a 254 nm Hg-vapour lamp and a 222 nm KrCl excimer lamp. In contrast to the mercury and excimer lamps, the emission wavelength peak of semiconductor-based UVC LEDs can be tuned in order to obtain the optimal compromise between limiting penetration depth to avoid DNA damage and providing sufficiently high irradiances for UVC deactivation of MRSA even in the presence of UVC-absorbing soil load. Due to their small form factors UVC LEDs can be easily arranged in larger arrays of any shape and size allowing the controlled irradiation of specific areas of skin without damaging the surroundings. Since the footprint of an UVC LED chip is typically 1 mm^2^ or less with a few hundred µm in thickness, compact UVC LED sources could also enable access to narrow body openings, e.g. in the nasal cavity or urinary tract in the future.

## Results

### Inactivation of MRSA

#### Persistence

To ensure a minimum number of colony forming units per test specimen, the recovery rates were determined. Recovery rates were obtained for 0.03% albumin sodium chloride solution. For MRSA DSM 11822 it was about (42 ± 6.4)% and for *S. epidermidis* DSM 1798 (30 ± 30)%. Higher rates were found for MSSA DSM 799 (68 ± 20)%. All tests were repeated three times. After drying, a bacterial burden of 1.5 × 10^5^–1.5 × 10^6^ colony forming units/germ carrier was achieved.

#### Bactericidal efficacy

**Figure 1:**
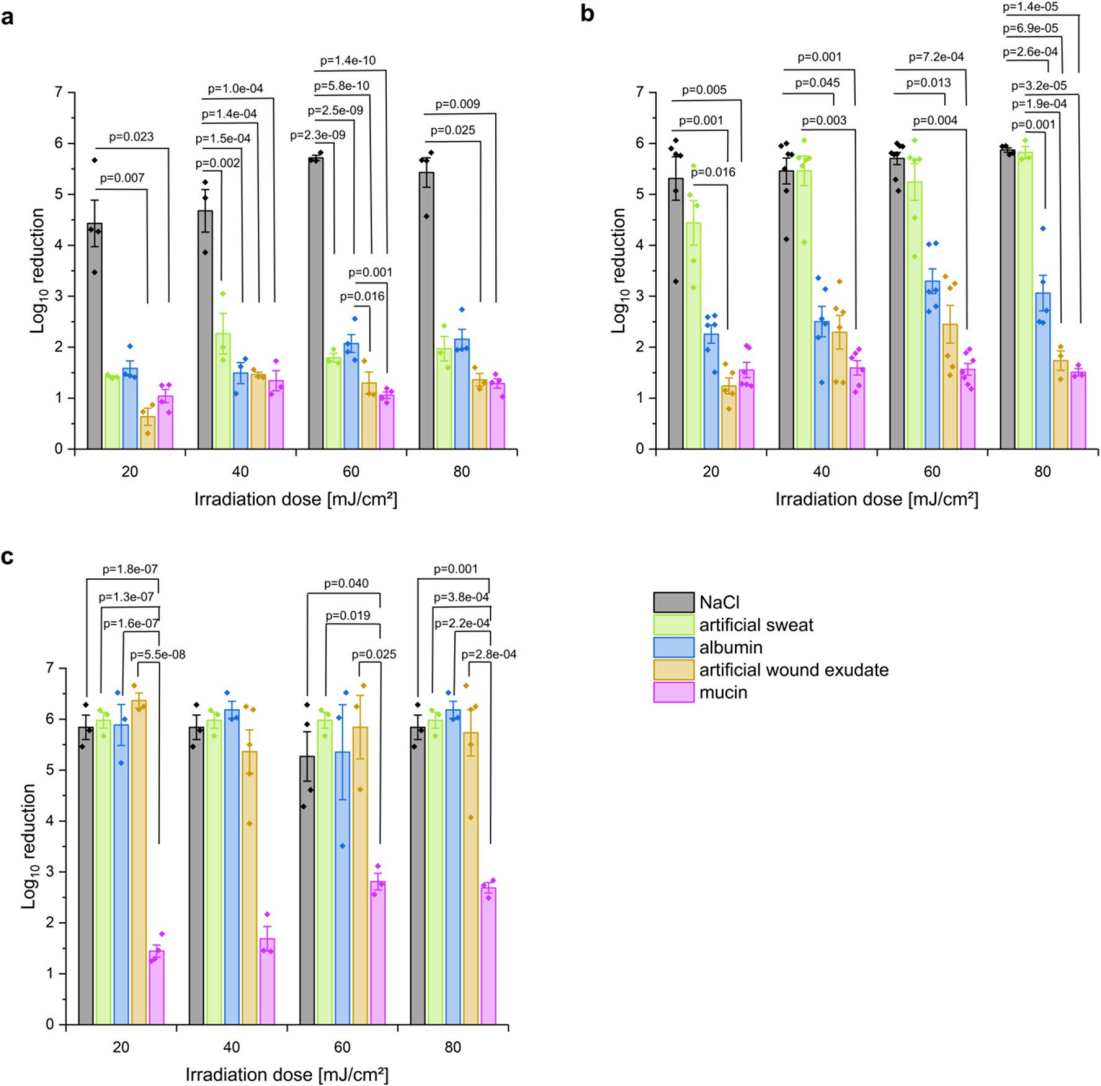
MRSA DSM 11822 log 10 reduction of colony forming units (cfu)/germ carrier for irradiation with 222 nm (**a**), 233 nm (**b**) and 254 nm (**c**). Bacteria were suspended in NaCl (black), artificial sweat (green), albumin (blue), artificial wound exudate (orange) and mucin (purple) solutions without and with soil load and dried for 30 min before irradiation. The presented p-values are based on one-way ANOVAs and Kruskal–Wallis tests followed by pairwise post hoc tests with Bonferroni correction. The data show mean ± SEM. *n* = 3–8.

The bactericidal efficacy of UVC irradiation with three different wavelengths and four doses, each, were proofed in a quantitative germ carrier test following DIN EN 14561^26^ and ASTM E2111-12^27^. To mimic realistic conditions, a number of soil loads were applied. Using UVC radiation of 222 nm (Fig. 1a), a log_10_ reduction (LR) of 4.4 was attained in sodium chloride solution with a dose of 20 mJ/cm^2^. Under soil load (artificial sweat pH 8.4, albumin 0.3 g/l, artificial wound exudate, mucin 0.5%), the LRs varied between 0.64 and 1.59. Increasing the dose from 20 to 40 mJ/cm^2^ led to a slight increase in bacterial reduction. Further rising irradiation doses did not result in an additional increase in efficacy.

Radiation with 254 nm wavelength with an irradiance of 0.29 mW/cm^2^ resulted in a strong reduction of viable microorganisms in sodium chloride solution, artificial sweat, albumin and artificial wound exudate (LR 5.84–6.37). For mucin, LRs of 1.45 at a dose of 20 mJ/cm^2^ up to 2.69 for a dose of 80 mJ/cm^2^ could be achieved (Fig. 1c).

In sodium chloride solution, reduction after irradiation at 233 nm was comparable to 254 nm (LR ≥ 5). With higher doses, the LR is rising slightly from 5.3 (20 mJ/cm^2^) to 5.7 (60 mJ/cm^2^) (Fig. 1b). Irradiation of bacteria in artificial sweat with a dose of 20 mJ/cm^2^ decreased the LR to 4.44. Doses higher than 20 mJ/cm^2^ reduced the applied microorganisms with an LR > 5. The lowest LRs were found with 0.03% sodium chloride albumin mixture (2.26–3.3), artificial wound exudate (1.24–2.45) and 0.5% mucin (1.51–1.59), whereby the LR was nearly constant with rising doses.

**Figure 2:**
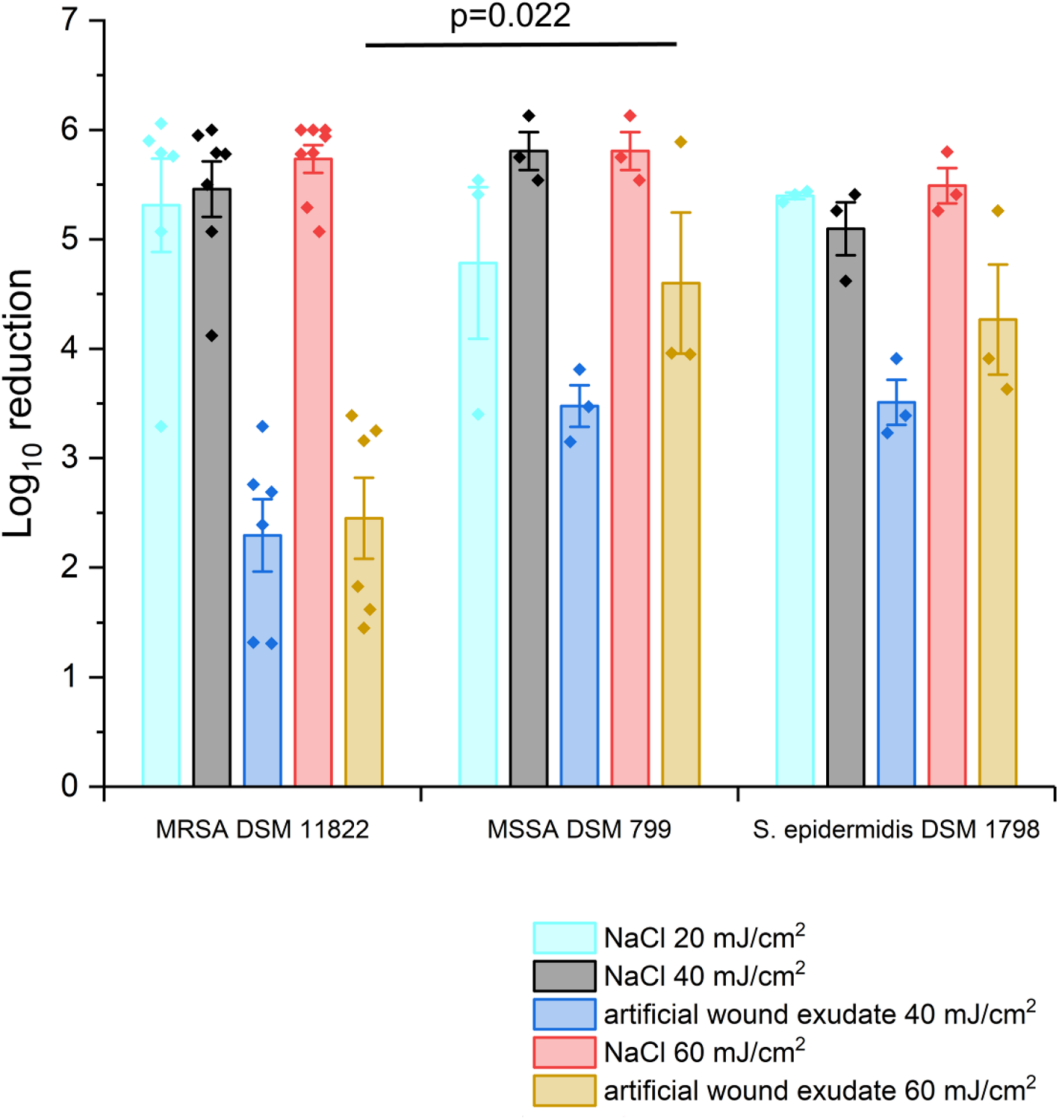
Log 10 reduction after treating different MRSA DSM 1182 and MSSA DSM 799 strains as well as *S. epidermidis* DSM 1798 with doses of 40 mJ/cm^2^ and 60 mJ/cm^2^ at 233 nm. Microorganisms were suspended in sodium chloride solution (20 mJ/cm^2^, cyan; 40 mJ/cm^2^, black; 60 mJ/cm^2^, red) or artificial wound exudate (40 mJ/cm^2^, blue; 60 mJ/cm^2^, orange) before drying on germ carriers for 30 min following irradiation. Multiple mean value comparisons were conducted to check for differences between the strains. The presented p-value is based on a Kruskal–Wallis test followed by pairwise post hoc tests with Bonferroni correction. The data show mean ± SEM. *n* = 3–8.

Comparing the inactivation efficacy of MRSA DSM 11822, MSSA DSM 799 and *S. epidermidis* DSM 1798 without adding a soil load (sodium chloride solution 0.9%), the irradiation at 233 nm did not result in significant differences (LR = 4.8–5.8), when irradiated with 20 mJ/cm^2^, 40 mJ/cm^2^ or 60 mJ/cm^2^. In artificial wound exudate, MRSA DSM 11822 is significantly less susceptible to irradiation at 233 nm (Fig. 2) than MSSA DSM 799 and *S. epidermis* DSM 1798 (40 mJ/cm^2^, 60 mJ/cm^2^). MRSA DSM 11822 was reduced by LRs of 5.5 and 5.7 at 40 and 60 mJ/cm^2^ in sodium chloride solution. Suspension in artificial wound exudate decreased the efficacy to LRs of 2.3 and 2.5. For MSSA DSM 799, the LR was 5.8 at an irradiation with 40 and 60 mJ/cm^2^. In artificial wound exudate, the LR was 3.5 at 40 mJ/cm^2^ and 4.6 at 60 mJ/cm^2^. *S. epidermidis* DSM 1798 was reduced by an LR of 5.1 and 5.5, respectively, in sodium chloride solution. In artificial wound exudate, the LR decreased to 3.5 and 4.3.

Absorption of soil loads differed clearly. At 230 nm, absorption was highest for mucin and the artificial wound exudate. For 222 nm the absorption was even higher. Sodium chloride solution has the lowest observed absorption at the relevant wavelengths (Supplementary Information, Fig. S1).

### Risk assessment

#### Cell viability

The cell viability was investigated directly after irradiation at different UVC wavelengths using an MTT test on punch biopsies of reconstructed epidermal human skin equivalents (RHEs) based on a fluorescence assay in accordance to^28,29^ (S1, Fig. S2). RHEs incubated for 1 h in sodium dodecyl sulphate (SDS, positive control) showed (2.1 ± 0.5)% viability. None of the applied doses resulted in a decrease of cell viability below 80%. Due to the photosensitivity of the cell culture medium, the RHEs were placed into PBS during irradiation. In order to verify if the minor reduction of cell viability was due to irradiation or the absence of medium, a non-irradiated RHE was kept for 30 min in PBS at room temperature (*n* = 1), which only showed a reduction to 96% cell viability (data not shown).

#### DNA damage

For DNA damage, CPDs and 6-4PPs were investigated after irradiation of excised human skin and RHEs, which is well suitable for studies of DNA damage and radical formation in skin during or after UV irradiation^30^.

**Figure 3:**
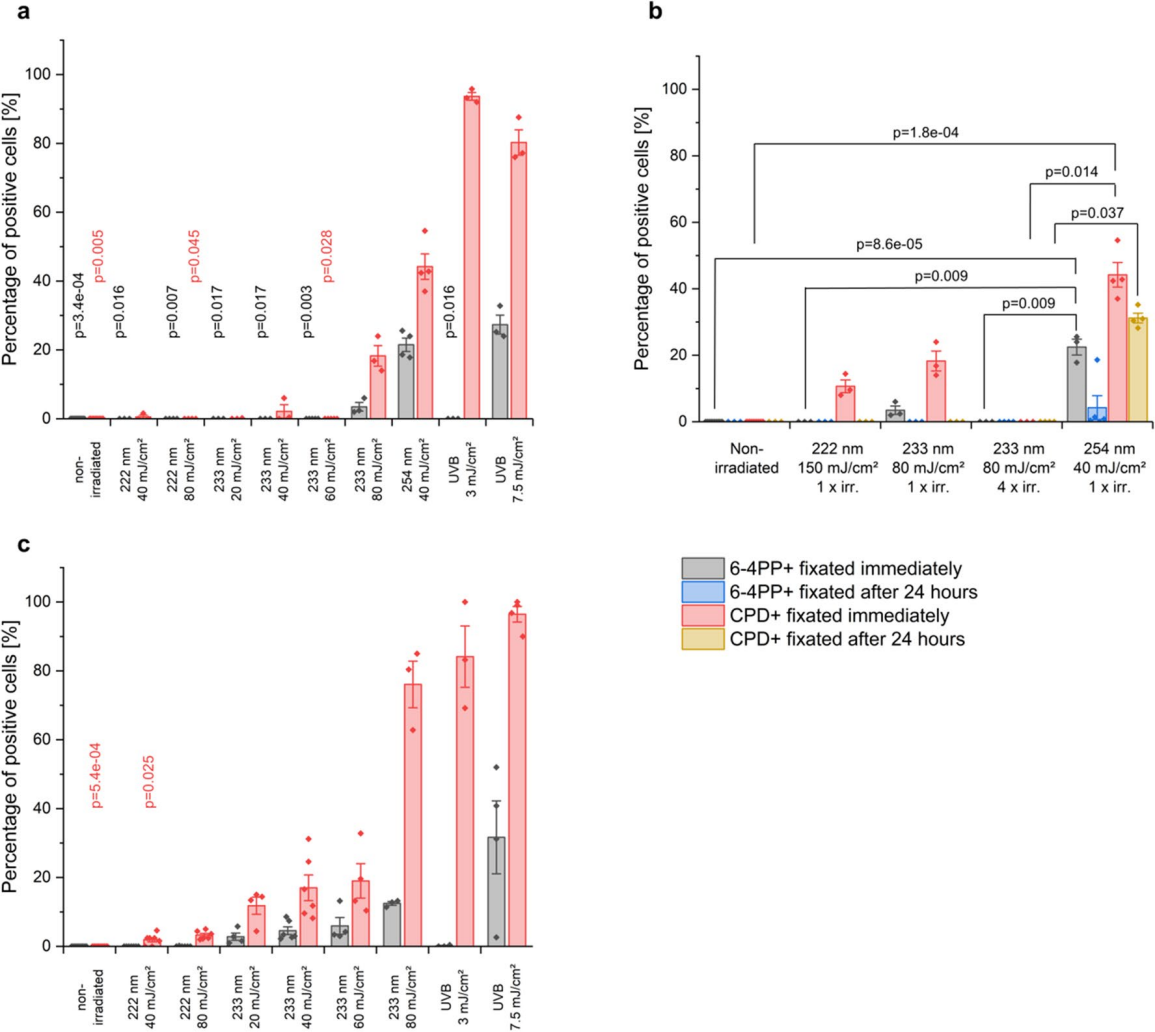
DNA damage of RHEs fixated directly (**a**) and 24 h after UV irradiation (**b**) and excised human skin (**c**). The data show the percentage of positive cells with 6-4PP (grey, blue) and CPD damage (red, orange). For both skin models, non-irradiated skin (negative control) shows no damage. In RHEs 44.2% of epidermal cells irradiated with 40 mJ/cm^2^ at 254 nm (positive control) show CPDs and 21.5% of the epidermal cells show 6-4PPs. In excised human skin, irradiation with 3 mJ/cm^2^ of UVB (positive control) induced 84.1% CPD positive cells. A reduction in DNA damage of RHEs was observed for fixation 24 h after irradiation with 40 mJ/cm^2^ at 254 nm (positive control), indicating a DNA repair mechanism (**b**). The CPD damage after 150 mJ/cm^2^ irradiation with 222 nm disappeared when fixating 24 h after irradiation. The weak damage after 233 nm irradiation with 80 mJ/cm^2^ when fixated immediately, disappeared when fixating 24 h later. Consecutive irradiation at 80 mJ/cm^2^ of 233 nm every 24 h did not show any DNA damage. The presented p-values derive from multiple comparisons with Bonferroni corrections after a Kruskal–Wallis test. Every group was compared to the positive control (**a**,**c**) and additionally the influence of multiple irradiations and fixation time was checked (**b**). The data show mean ± SEM. *n* = 3–12.

**Figure 4:**
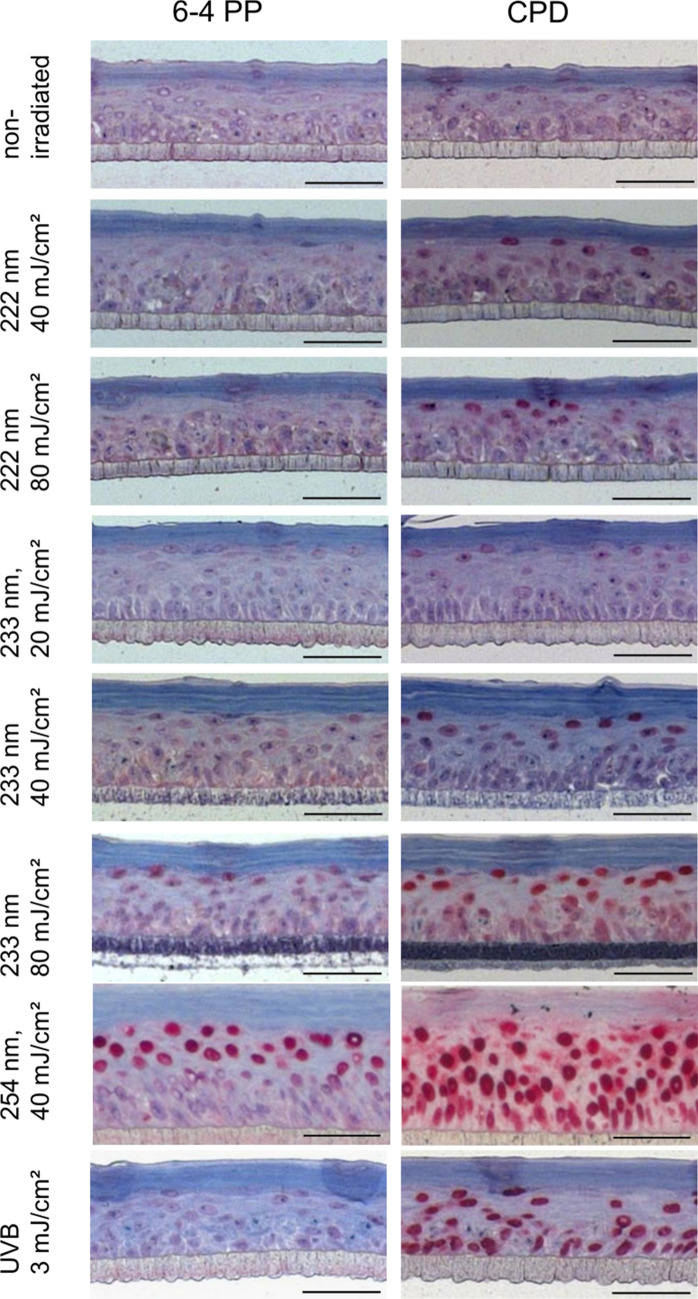
Representative images of histological analysis of DNA damage in RHEs. Paraffin sections are stained for 6-4PP (left column) and CPD damage (right column). Positive cells are stained in dark red. Non-irradiated RHEs (negative control) show no positive cells, while positive epidermal keratinocytes of RHEs irradiated with 40 mJ/cm^2^ at 254 nm (positive control) were found even in the deeper layers of the epidermis. RHEs irradiated with 3 mJ/cm^2^ UVB (0.1 MED) showed > 90% CPD damage throughout the whole epidermis. The CPD damage after 40 mJ/cm^2^ irradiation at 222 nm and 233 nm only occurred at the uppermost layer of the epidermis. Scale bar: 50 µm.

As positive control, irradiation of 40 mJ/cm^2^ at 254 nm resulted in (21.5 ± 1.9)% 6-4PP and (44.2 ± 3.7)% CPD positive epidermal keratinocytes, while untreated RHEs as negative control showed no damage (6-4PP: *p* = 3.4e−04, CPD: *p* = 0.005) (Figs. 3a, 4).

Doses of 20–60 mJ/cm^2^ at 233 nm resulted in only negligible CPD damage which occurred only on the superficial layer of the epidermis (Fig. 4). When raising the dose to 80 mJ/cm^2^, the CPD damage increased to (18.3 ± 3.0)%. Irradiation of 222 nm at 40 mJ/cm^2^ resulted in (0.5 ± 0.5)% CPD positive keratinocytes; DNA damage after irradiation at 80 mJ/cm^2^ was not observed (6-4PP: *p* = 0.007, CPD: *p* = 0.045). Similar to the irradiation at 233 nm, this damage was limited to the superficial layer of the epidermis (Fig. 4).

Broad band UVB irradiation (280–400 nm) with a dose of 3 mJ/cm^2^, which amounts to ≈ 10% of a minimal erythema dose (MED) for skin type II, provoked (93.7 ± 1.1)% CPD keratinocytes (Fig. 4). All damage caused by the high energy UVC is located very superficially directly below the SC (Fig. 4), whereas 254 nm and UVB-induced damage reaches down to the basal cells.

The maximal depth of DNA damage observed in the epidermis of RHE was measured on 5 positions of the histological images from the skin surface (Supplementary Information, Fig. S3a). The measured SC thickness was subtracted on every position. For 40 mJ/cm^2^ at 254 nm, CPD and 6-4PP damage was observed in the entire epidermis and part of the dermis including the vulnerable basal cells. Similar CPD damage depths were detected after UVB irradiation at ≥ 3 mJ/cm^2^; the 6-4PP damage at the higher dose occurred down to (3.2 ± 1.0) µm depth. In contrast, at 222 nm irradiation, CPD damage depths of maximally (10.7 ± 3.7) µm were observed for 150 mJ/cm^2^. For irradiation at 233 nm, maximal depths of CPD damage of (16.1 ± 0.7) µm were observed for 80 mJ/cm^2^. The epidermal thickness was determined to be 37–50 µm.

The mean SC thickness of RHEs was (17 ± 4.3) µm. An increase after single or multiple irradiation could not be observed. The thickness of the epidermis only increased insignificantly after fourfold irradiation on four consecutive days from (95.6 ± 2.9) µm for non-irradiated, to (97.4 ± 0.9) and (97.8 ± 3.5) µm for RHEs irradiated with 60 and 80 mJ/cm^2^ at 233 nm, respectively (each *n* = 4).

As shown in Fig. 3b, the CPD and 6-4PP damage measured for the RHEs immediately after irradiation with 40 mJ/cm^2^ at 254 nm (positive control) partly regenerated after their incubation in medium for 24 h. After irradiation with 150 mJ/cm^2^ at 222 nm, the (10.7 ± 1.9)% CPD damage disappeared when the RHEs were fixated 24 h after irradiation.

For 80 mJ/cm^2^ irradiation at 233 nm, the immediately determined CPD damage disappeared during 24 h of further incubation. In order to evaluate a possible accumulative effect, the measurements were repeated after multiple irradiations. RHEs were irradiated four times every 24 h with the identical dose and incubated in medium at 37 °C in between. No DNA damage was observed at 80 mJ/cm^2^ at 233 nm (Fig. 3b). Non-irradiated control RHEs were incubated for the entire time with daily breaks for 30 min, showing no DNA damage. Selected investigations using cleaved caspase-3 staining for apoptosis^31^ showed no apoptosis positive cells of RHEs fixated after 24 h for 40 mJ/cm^2^ at 254 nm (*n* = 4), 150 mJ/cm^2^ at 222 nm (*n* = 3), as well as 60 (*n* = 4) and 80 mJ/cm^2^ (*n* = 4) at 233 nm, as well as for non-irradiated RHEs (*n* = 3) (data not shown).

**Figure 5:**
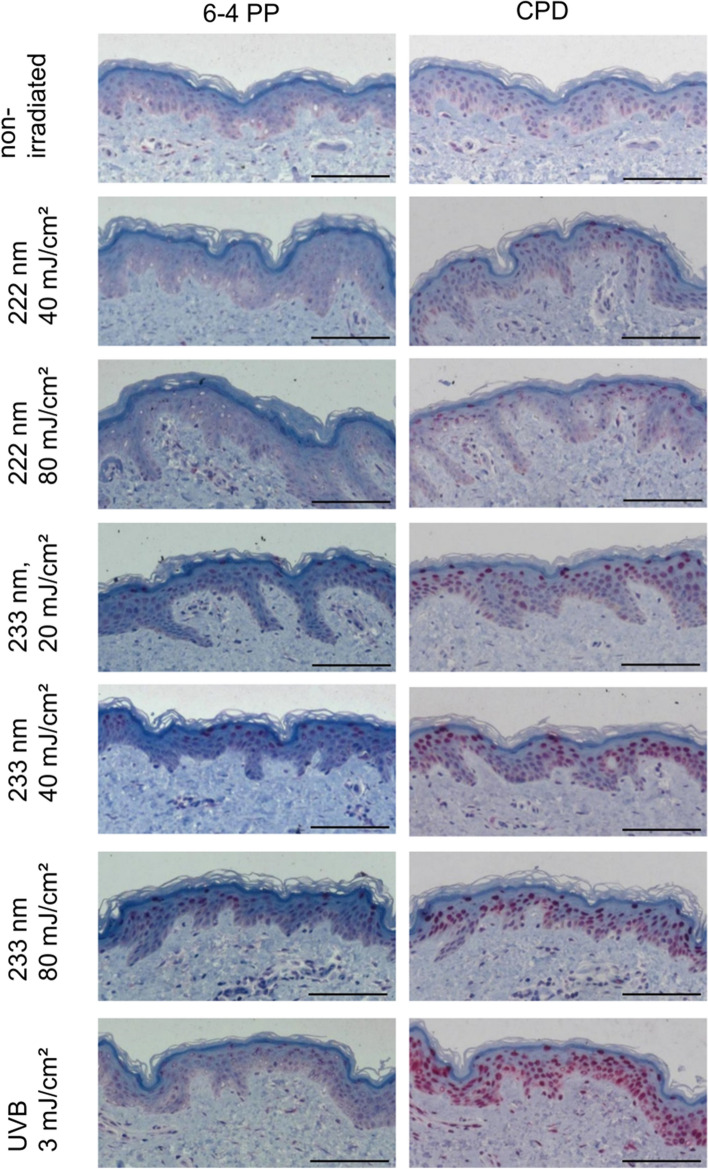
Representative images of histological analysis of DNA damage in excised human skin. Paraffin sections are stained for 6-4PP (left column) and CPD damage (right column). Positive cells are stained in dark red. Non-irradiated human skin (negative control) shows no positive cells. Human skin irradiated with 3 mJ/cm^2^ UVB (0.1 MED) showed > 80% CPD damage throughout the whole epidermis. The CPD damage after 40 mJ/cm^2^ irradiation at 222 nm and 233 nm only occurred on the uppermost layer of the epidermis. Scale bar: 100 µm.

Additionally to RHE irradiation, experiments were also performed on excised human skin obtained from plastic reduction surgeries. Here, UVB (280–400 nm) at 3 mJ/cm^2^ (≈ 1/10 MED) was applied as a positive control leading to (84.1 ± 8.9)% CPD damage, while non-irradiated skin showed no DNA damage (*p* = 5.4e−04) (Figs. 3c, 5).

Irradiation with 222 nm led to maximally (3.3 ± 0.5)% CPD damage at 80 mJ/cm^2^ (*p* = 0.045). For irradiation at 233 nm, a gradual increase of CPD damage from (11.8 ± 2.5)% (20 mJ/cm^2^) to (76.1 ± 5.6)% (80 mJ/cm^2^) was observed.

The mean SC thickness of excised human skin was (12.5 ± 2.1) µm. The DNA damage depth for ≥ 3 mJ/cm^2^ UVB irradiation, was observed in the entire epidermis down to ≥ 30 µm (Supplementary Information, Fig. S3b). At 222 nm irradiation, a maximal depth of CPD damage of (4.6 ± 0.3) µm was observed for 150 mJ/cm^2^. For 80 mJ/cm^2^ irradiation at 233 nm, a maximal CPD damage depth of (16.8 ± 1.6) µm was determined.

#### Radical formation

**Figure 6:**
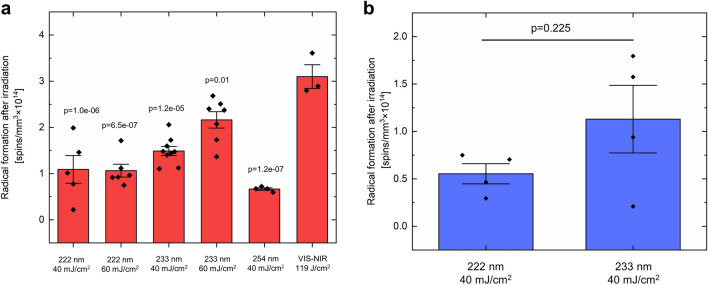
Radical formation of RHEs (**a**) and excised human skin (**b**) after UV irradiation. The data show the spin concentration (spins/mm^3^ × 10^14^) representative for radical formation in RHEs (**a**) and excised human skin (**b**) after irradiation with different wavelengths and irradiation doses. The corresponding *p*-values were obtained by comparing the different UVC wavelengths against VIS–NIR by one-way ANOVA with Dunnett post hoc tests for RHEs (**a**). All groups showed significantly lower radical formation in comparison to VIS–NIR. In excised human skin (**b**), the radical formation after irradiation with 233 nm (40 mJ/cm^2^) was twofold higher compared to irradiation with 222 nm (40 mJ/cm^2^). Radical formation was compared with the Student’s *t*-test for paired samples and showed no significant differences. Data were collected with *n* = 3 to *n* = 9 RHE models and *n* = 4 donors of excised human skin with 2 to 3 technical replicates, each. The data show mean ± SEM.

EPR spectroscopy was used to assess the radical formation after irradiation. For biological assessment of the formed radicals, irradiation in the visible and near-infrared spectral region (λ = 400–2000 nm) was performed for 20 min (Dose: (118.7 ± 0.8) J/cm^2^), which is considered to be harmless. This dose induced a radical formation of (3.10 ± 0.26) × 10^14^ spins/mm^3^. A lower radical formation of maximally (1.09 ± 0.30) × 10^14^ spins/mm^3^ was detected after irradiation of RHEs with 222 nm of the doses 40 mJ/cm^2^ or 60 mJ/cm^2^ (*p* = 1.0e−06 and *p* = 6.5e−07). Furthermore, also an irradiation with 254 nm (40 mJ/cm^2^) induced a lower radical formation of (0.67 ± 0.03) × 10^14^ spins/mm^3^ (*p* = 1.2e−07). A dose of 40 mJ/cm^2^ and 60 mJ/cm^2^ of 233 nm induced an average spin concentration of (1.49 ± 0.10) × 10^14^ spins/mm^3^ and (2.16 ± 0.18) × 10^14^ spins/mm^3^, which was 52% and 29% lower than the irradiation with 119 J/cm^2^ visible-near infrared (VIS–NIR) (Fig. 6a, *p* = 1.2e−05, *p* = 0.01), respectively.

Radical formation on excised human skin was only investigated for 40 mJ/cm^2^. The radical formation in ex vivo human skin is presented in Fig. 6b. A radical formation of (0.55 ± 0.11) × 10^14^ spins/mm^3^ was observed after irradiation of excised human skin with 222 nm. Irradiation with 233 nm induced a twofold higher average spin concentration of (1.13 ± 0.36) × 10^14^ spins/mm^3^ (*p* = 0.225).

## Discussion

### Inactivation of MRSA

Conventionally, UV radiation with 254 nm wavelength is used for surface decontamination^32^. The radiation inactivates bacteria by damaging DNA, RNA and proteins resulting in, e.g. DNA breakage due to the energy received from absorption. Maximum absorption takes place at wavelengths lower than 300 nm with a second minor minimum at 230 nm. Additionally, especially *S. aureus* strains are susceptible to UV radiation because of their higher content of thymine bases, leading to CPD as well as 6-4PP damage^33^. This suggests a biocidal efficacy of 233 nm radiation against MRSA as well as MSSA.

However, all microorganisms harbour different repair mechanisms as base excision repair, UV damage endonucleases or nucleotide excision repair to treat mutations or even DNA strand breaks. One mechanism is the photo reactivation by photolyases using energy of blue light to repair damage, which primarily results from UV irradiation as CPD and 6-4PP damage^34–36^. Maclean et al.^34^ stated a degree of photo reactivation of up to 0.025 for *S. aureus*. Another mechanism of *S. aureus* to repair damage resulting from UV irradiation is a special SOS-response that includes RecA and LexA, two hypothetical proteins. Cirz et al.^37^ created a mutant strain lacking LexA that was more sensitive to UV treatment than the wild-type strain. But even if bacteria have various repair mechanisms, since UVC radiation is almost absent in the environment, bacteria do not have any direct protection against it. That is why no differences in efficacy between different species or strains were expected. On the other hand, MRSA have evolved different mechanisms to resist treatment with antibiotics^4^. These resistance mechanisms comprise a set of DNA repair mechanisms as well as mechanisms for detoxification of, e.g. reactive oxygen species, additionally to those evolved in MSSA. These mechanisms might increase the tolerance to UV radiation as well. Indeed, in the presented study, a lower sensitivity of the MRSA strain DSM 11822 in comparison to the MSSA strain DSM 799 and *S. epidermidis* DSM 1798 was observed for irradiation at 40 mJ/cm^2^ and 60 mJ/cm^2^ in artificial wound exudate. But, in sodium chloride solution, no significant differences were observed, even when irradiated with lower doses. This might be due to the nearly full inactivation of bacteria. Similar results were obtained by Kerr et al.^38^, who treated MRSA and MSSA plated on blood agar plates with various high doses of UV light (254 nm) and observed no differences in reduction between the strains.

Without the addition of a soil load, e.g. albumin or inorganic compounds, the newly developed 233 nm UVC LED source was able to reduce the viable microorganisms by more than an LR of 5, thus reaching the required reduction as stated in various EN standards for disinfection and antisepsis^26,27,39^. The soil loads added are substances naturally occurring in saliva as mucin or proteins (albumin) or sweat (different salts) or wound exudate (proteins) to mimic realistic conditions.

However, the existence of organic and inorganic loads in the natural habitat of the microorganisms, as it exists on the skin or the mucosa, decrease the biocidal efficacy of radiation by absorption of photons. Nevertheless, the used doses in the presented study to investigate the biocidal activity of radiation at 233 nm were limited to a maximum of 80 mJ/cm^2^ with regard to skin compatibility. Indeed, decreasing biocidal effects were clearly observed for all tested wavelengths when adding a soil load. Especially mucin, a biopolymer as part of the mucus, which is present also on the surfaces of the mucosa in the human body, had protective effects. At 233 nm, the protection by albumin was comparable to that of mucin. In contrast, inorganic salts as found in human sweat certainly had a protective effect, but this effect was overcome by doses of > 40 mJ/cm^2^. Interesting, that the reduction factors at 233 nm when using mucin or proteins are not increasing with increasing doses. A reason might be the strong absorbance of proteins and mucin at the given wavelengths resulting in a protection of the bacteria below. At 254 nm, reduction factors when using mucin are slightly increasing with increasing doses, possibly resulting from the higher penetration depth of the radiation. However, the differences are small.

Interestingly, the addition of soil loads led to a reduction of viable microorganisms with nearly equal LR, independent of the applied dose. Similar effects were observed for radiation with 222 nm. A reason might be the reduced penetration depth of radiation with lower wavelength. Soil loads might act as a shield, protecting the bacteria. This corresponds to the protection effect of living cells of the skin. The slightly higher LR of 233 nm compared to 222 nm supports this assumption. In the Supplementary Information, Fig. S1, the absorption spectra of selected soil loads are shown. Sweat, which almost does not reduce the LR for 233 nm, provides very low absorption at this wavelength but the absorption is increased at 222 nm and thus, decreases the LR here. Mucin also shows an absorption at all wavelengths but it is less pronounced at 254 nm, which could explain the reduced LR at 254 nm but the increase with dose.

As stated in various EN standards, an LR ≥ 5 is necessary for disinfection/antisepsis^26,27,39^. However, even lower biocidal effects with an LR between 1 and 3, which means a reduction of 90.0–99.9% of viable microorganisms, are able to reduce the risk of infection.

Similar to absorption processes by proteins added as soil load, also extracellular substances, produced by biofilm-forming bacteria as well as clusters of microorganisms, can absorb the radiation leading to reduced biocidal effects^40^. Next to liquid layers as saliva or sweat, in clinical practice also biofilms with a certain thickness may occur. Even if these biofilms will be removed before treatment by rinsing with, e.g. sodium chloride solution, the bacterial load will be high resulting in multi-layered bacterial burden that possibly shields bacteria in the lower layers. To consider these factors, the bacterial load in the laboratory experiments was high (10^7^ cfu/ml) and further soil loads as mucin and salts were added. However, to what extent the treatment of biofilms, e.g. as found in wounds, with radiation of 233 nm is efficient for antisepsis has to be clarified in further studies.

Another point is the higher bactericidal effect when the irradiance of the 254 nm lamp was raised. Higher irradiances at 233 nm might also lead to higher reduction of viable microorganisms in solutions containing organic or inorganic soil loads. For better understanding of the relationship between dose, irradiance and bacterial eradication, and also to determine the optimal treatment procedure for antimicrobial efficacy, further investigations are necessary.

### Risk assessment

#### Cell viability

Due to the strong variability, it is generally accepted that only a decrease of cell viability < 80% is considered meaningful^41,42^. In our experiments, this threshold was not reached. Thus, in the investigated time frame the viability was not significantly reduced, indicating that the cell viability was not affected directly by irradiation. It cannot be excluded that cell viability would decrease at later time points. During irradiation, the RHEs were kept in PBS instead of medium as UV interacts with the medium, entailing death of keratinocytes due to the formation of ROS via riboflavin photosensitisation^43^.

#### DNA damage

The applied doses of far UVC irradiation (222 and 233 nm) provoke only a small fraction of the CPD damage generated by 10% MED of UVB radiation. This dose is at a level likely unavoidable in daily life.

Although there is no threshold of UV exposure, under which it is considered to be harmless^44,45^, it must be argued that a low amount of UV exposure is necessary for vitamin D generation^46^. The pre-vitamin D concentration reaches a maximum at a dose far below 1 MED. The low amount of DNA damaged by far-UVC irradiation is located directly below the SC. No damage is found in the vicinity of the basal cells, which are most vulnerable, whereas the applied doses of UVB and 254 nm irradiation induce DNA damage throughout the entire epidermis and reach the fibroblasts in the dermis. The results indicate that 233 nm penetrate deeper than 222 nm, which is comparable for RHEs and excised human skin. Thus, the DNA damage at the required dose to reduce the pathogens (40–60 mJ/cm^2^) by an LR of 5 using high energy UVC can be neglected.

Compared to irradiation with 233 nm at 80 mJ/cm^2^, the CPD formation was only 2.5-fold higher after irradiation with 254 nm (40 mJ/cm^2^), while 0.1 MED of UVB induced a fivefold higher damage. It has to be mentioned that the irradiation with 254 nm had to be repeated in the framework of the experiments. Since the RHEs used for 254 nm showed a higher SC thickness (28.1 ± 0.8 µm) compared to the samples used for 222 nm, 233 nm and UVB, the observed DNA damage after irradiation with 254 nm might be underestimated.

#### Repair mechanisms

The DNA damage induced by UV exposure is not persistent and can quickly be reversed by the enzymatic repair system^47^ or apoptosis^48^.

Experiments with increased regeneration times were performed on RHEs. As for the previous experiments, half of the RHEs were fixated directly after irradiation, the other half were set in medium and incubated for further 24 h at 37 °C, 5% CO_2_. Here, a complete repair of DNA damage was observed within 24 h after high energy UVC irradiation at 233 nm. Previous studies using 222 nm suggested a repair mechanism for CPD damage after 7 days^9^. While the molecular dynamics for CPD and 6-4PP damage have been described to occur as fast as in the femtosecond range^49^, the repair mechanisms in irradiated skin became effective within days. Using broad band UV irradiation (43% UVA, 54% UVB and 3% UVC), a significant reduction of CPD damage was observed 3 days after irradiation in human skin in vivo, which further reduced to zero after 10 days^45^. In another study, most initial CPD damage was repaired within 48 h after UV irradiation (95% UVA, 5% UVB) with 80% MED^44^. In another study using hairless mice (Hos:HR-1) and 222 nm irradiation, the CPD damage was gradually reduced from 37 to 13% within 24 h^50^.

Apoptosis was not observed 24 h after RHE irradiation at the selected doses. Nevertheless, we cannot exclude the formation of other photoproducts which are described for example for hydrolysed keratin in the UVB spectral range^51^, or other degradation processes such as epidermal CXCL5 mRNA and protein expressionn^52,53^. Up regulation of epidermal CXCL5 was independent of keratinocyte differentiation and keratinocyte-keratinocyte interactions in epidermal layers.

Upon multiple irradiations, our results suggest that the process of DNA damage formation restarts after every irradiation, if the RHEs can fully recover. The (18.5 ± 3.9)% CPD and (1.1 ± 1.0)% 6-4PP damage for RHEs fixated 24 h after single irradiation (80 mJ/cm^2^ at 233 nm) vanished after irradiating four times every 24 h with identical fixation. Therefore, it can be concluded that the repair mechanism becomes more effective after the first 24 h. Otherwise, no decrease in DNA damage would be expected. In other studies, the occurrence of sunburn and desquamation was observed in dorsal skin of mice irradiated at 254 nm, but not at 222 nm after 4 days^50^. We did not observe this effect in RHEs. Neither was an increase of the epidermal thickness observed. CPDs are considered to be primarily related to the formation of skin tumours due to the quicker repair of 6-4PPs^54,55^. In this study, we only observed 6-4PP damage > 20% if the CPD damage was > 90%. This can be explained by a relatively higher formation of CPD damage, but also a quicker repair is possible, which occurs already in the timeframe between irradiation and fixation (≈ 3 min).

#### Excised human skin

Our experiments using excised human skin followed the same trend, but resulted in considerably more DNA damage and variation compared to RHE models after irradiation with 233 nm. At 40 mJ/cm^2^ 233 nm irradiation, excised human skin showed tenfold increased CPD damage compared to RHEs.

The larger variations for excised human skin can be explained by increased biological variability. The higher DNA damage could be related to the lower enzymatic activity of repair enzymes, such as photolyase. Human skin must be transported from the surgery to the laboratory and was stored for max. 24 h at 4 °C without culture medium supply. A comparison with a previous investigation^24^ showed that the DNA damage of porcine ear skin was in between that of RHEs and excised human skin. Similar storing conditions to excised human skin were conducted in this case. The different SC thickness could also contribute to this effect. The irradiated RHEs had 30% lower SC thickness than the obtained human skin. The SC thickness determined by histological sections is influenced by the procedure of staining, but this influence should be identical for both models. As shown by the non-irradiated control, pre-existing DNA damage or effects of surgical disinfection can be ruled out.

#### Radical formation

It could be shown that radical formation was measurable but significantly lower at 233 nm irradiation than after irradiation with VIS–NIR on RHE. In human skin no significant difference between 233 nm irradiation and 222 nm irradiation could be found, which is correlated to the relatively high data spread within the 233 nm group. This might be based on the diversity in human skin type, respectively melanin and antioxidant content. To elucidate this phenomenon in more detail, further studies with higher number of samples are required.

The assessment criteria of how many and which radicals are physiological and where the detrimental turning point is, are more uncertain than for DNA damage but 20 min VIS and NIR irradiation could be considered to be safe and thus the radical formation during this exposure as well. Furthermore, we could show a higher radical formation in RHEs for 233 nm as well as for 222 nm (40 mJ/cm^2^) as compared to ex vivo human skin (32% and 98%). These findings correspond with the statement of Albrecht et al.^30^ that the radical formation in reconstructed human skin is higher compared to ex vivo skin when irradiated by a simulated sun spectrum (305–2200 nm). The observed effect can be explained by a lasting metabolic rate within RHEs^30^ during irradiation as the RHEs are more metabolically active than excised human skin and contain less antioxidants because no exogenous antioxidants are available. In the latter study, the radical load of reconstructed human skin was comparable to the in vivo situation. However, all these investigations have to be confirmed in vivo for UVC irradiation.

## Conclusion

The results of our investigations pave the way for future therapies to decolonise patients with MSSA and MDR bacteria such as MRSA in various areas (nasal cavity, throat, wounds) in order to prevent infections as well as the spread of MDR in hospitals. Therefore, further in vitro and in vivo studies are planned to assess the impact of 233 nm UVC irradiation on various bacteria, biofilms, on human mucosa, skin and corneal biopsies. Furthermore, the use of 233 nm UVC in everyday clinical practice is conceivable to treat all patients after admission and thus significantly reduce the MRSA problem. It should also be examined whether other MDR besides MRSA can be effectively eradicated by this UVC radiation.

At the biocidal doses for multi-resistant pathogens at 40 to 60 mJ/cm^2^ irradiation of 222 and 233 nm irradiation, achieving an LR of 5, no relevant DNA damage and radical formation occurred in the skin.

The bacterial eradication efficacy demonstrated for the recently developed 233 nm LED may also suggest its application/suitability to inactivate SARS-Cov-2^56,57^; like it has been already shown for 254 nm^58^ and 222 nm^59^.

## Methods

### Microorganisms, media, and bacterial multiplication

As test organisms, the Gram-positive bacteria *S. aureus* DSM 799 (ATCC 6538) and DSM 11822 as well as *S. epidermidis* DSM 1798 were used. *S. aureus* DSM 11822 is a MRSA strain, whereas DSM 799 is a MSSA strain.

Cryopreserved bacteria were inoculated onto Columbia blood agar plates and incubated for 24 h at 37 °C as a first subculture. After verifying the purity of the strains by examination of visible criteria as colour and colony shape, these subcultures were used as starting cultures for the following experiments. One colony was picked, plated on fresh TSA agar plates (Carl Roth, Karlsruhe, Germany), and incubated again at 37 °C for 24 h. The bacteria were harvested by rinsing the plate with 2 ml sodium chloride solution (0.9%) and the suspension was centrifuged three times at 10,000×*g* for 1 min and once at 7,150×*g*. The supernatant was discarded each time and the pellet resuspended in fresh sodium chloride solution. Afterwards, the pellet was resuspended in the relevant solutions used for further experiments. To ensure appropriate number of colony forming units (cfu) of 1–3 × 10^8^/ml, the optical density at 620 nm (OD_620_) was adjusted to 0.10–0.14 for *S. aureus* strains, to 0.30–0.35 for *S. epidermidis* and to 0.12–0.15 for *P. aeruginosa*. The bacterial suspensions were used within 2 h after adjusting the initial suspension to the appropriate density.

### Soil loads

In addition to bacteria suspensions in sodium chloride solution, different soil loads in sodium chloride solution were used. For protein load 0.03% albumin solution was used (Carl Roth, Karlsruhe, Germany) as minor load given by EN 14561 and EN 13727. For soil load of mucosa 0.5% mucin was used (Carl Roth, Karlsruhe, Germany). Artificial sweat pH 8.4 was composed of 0.5% NaCl solution with 0.05% l-histidine (Merck KGaA, Darmstadt, Germany), 0.5% Na_2_HPO_4_ (Carl Roth, Karlsruhe, Germany)^60,61^. Artificial wound fluid was composed of Eagle’s minimal essential medium with Earle’s salts with 2 mM l-glutamine (both PAN-Biotech GmbH, Aidenbach, Germany) and 10% fetal bovine serum (gibco Life Technologies, Carlsbad, CA, USA)^62,63^. Experiments for comparison of LRs by using different irradiances (254 nm) were carried out in cell culture medium (DMEM/F12, PAN Biotech GmbH, Aidenach, Germany) with addition of 10% fetal bovine serum (gibco Life Technologies, Carlsbad, CA, USA) and 2 mM l-glutamine.

### Drying on germ carrier and recovery rate

The initial suspensions with 1–3 × 10^8^ cfu/ml were further diluted from 1 × 10^7^ cfu/ml up to 10 cfu/ml. To confirm the correct number of cfu/ml, 100 µl of the suspensions likely containing 10^3^–10 cfu/ml were plated in duplicate on TSA plates; the colonies were counted after incubation at 37 °C for 24 h and the amount of cfu/ml at the starting solution was calculated. For further experiments, 100 µl of the suspensions containing likely 10^7^ cfu/ml were pipetted on the germ carrier (stainless steel, 20 mm diameter, polished to grade 2), spread with an inoculating loop, and dried for 30 min in a laminar flow cabinet inspired by EN 14561:2006 and ASTM E2197-11.

To ensure a number of minimum 1 × 10^5^ cfu/test specimen after drying, the recovery rate in sodium chloride solution after 30 min of drying was determined for 0.03% albumin sodium chloride solution. Specimens with dried bacterial suspension were placed in a 6-well-plate containing 3 ml of tryptone sodium chloride solution and Ø = (2.9–3.5 ± 0.3) mm glass beads per well. The specimens were incubated while shaking for 2 min (400 rpm) on an orbital shaker to detach the bacteria, and the resulting suspensions were diluted and plated on TSA plates in duplicate followed by incubation for 24 h at 37 °C to determine the number of viable bacteria. Simultaneously, 100 µl of the initial solution (1 × 10^7^ cfu/ml) were diluted in the same way and plated on agar plates without spreading on germ carriers before.

### UV irradiation

Irradiation of bacteria as well as excised human skin and RHEs was conducted with different UVC irradiation modules. UVC radiation of the wavelengths 254 nm (0.29 mW/cm^2^ for bacteria and 0.54 mW/cm^2^ for excised human skin and RHEs, LPL-R-01, Hg gas discharge lamp, sglux GmbH, Berlin, Germany, as positive control), 233 nm (0.041 mW/cm^2^, UVC LED irradiation source with a short pass optical filter suppressing wavelengths > 240 nm, Ferdinand-Braun-Institut, Berlin, Germany)^24^ and 222 nm (3.34 mW/cm^2^, ExciJet222 30–130 Kit (111073) Kr-Cl Excimer lamp with a short pass filter suppressing wavelengths > 230 nm, USHIO Deutschland GmbH, Steinhöring, Germany) were examined for their eradicating effect on different pathogens, and their effect on skin (cell viability, DNA damage, radical formation). Doses of 20, 40, 60 and 80 mJ/cm^2^ were investigated. The UVC LED irradiation system is comprised of an array of 120 LEDs with a narrow band emission peaking at a wavelength of 233 nm and a full-width at half maximum (FWHM) of 12 nm^25^. In order to remove unwanted emission at wavelengths > 240 nm, a short pass filter was added consisting of HfO_2_ and SiO_2_ quarter wavelength stacks that form a distributed Bragg reflector (DBR) with a photonic stopband between 240 and 300 nm. The spectrally pure 233 nm LED irradiation system delivered a uniform optical power density of 0.041 mW/cm^2^ over a target area of 70 mm × 70 mm at a distance of 25 mm from the system.

For detecting the microbicidal efficacy, all experiments were conducted with MRSA DSM 11822. Comparability of the effects with irradiation at 233 nm was investigated using MRSA DSM 11822, MSSA DSM 799 and *S. epidermidis* DSM 1798. Additionally, for the 254 nm UVC irradiation module, the efficacy of different doses by varying irradiance (0.45 mW/cm^2^, 0.9 mW/cm^2^) was proofed.

To assess skin DNA damage, a UVB lamp, containing also a small UVA fraction (280–400 nm, 0.041 mW/cm^2^; TH-1E; Cosmedico Medizintechnik, Stuttgart, Germany) was applied to induce 1/10 MED. For assessment of the radical formation, irradiation was additionally performed using a solar simulator (Low Cost Solar Simulator LS0104, with Lamp Type Xenon short arc 150W, LOT-Quantum Design GmbH, Darmstadt, Germany) with an optical fibre (liquid-filled, transmission: VIS/NIR (420–2000 nm) and a long pass filter (cut-on wavelength of 400 nm, 400FH90-50S, LOT-Quantum Design GmbH) as well as AMO Filter LSZ185 (air mass 0, outer space) to simulate solar irradiation within the VIS–NIR range on earth.

Irradiance of radiation and doses for 254 nm, 233 nm and 222 nm, were measured with the UV radiometer SXL55 with a SiC UVC sensor (sglux GmbH, Berlin, Germany), and an ILT 1400 Radiometer Photometer (International Light Technologies, Peabody, MA, USA) for UVA (SEL033) and UVB (SEL240) lamps. The irradiation dose for VIS–NIR was measured with an 843-R optical power meter and a 919-003-10 detector (Newport Spectra-Physics GmbH, Darmstadt, Germany).

### Irradiation of microorganisms on germ carriers/antibacterial efficacy of irradiation

After spreading and drying of the bacterial suspension on the germ carrier, the microorganisms were irradiated with varying doses of 222 nm, 233 nm and 254 nm (positive control) wavelength. Towards exposure, the specimens were treated as described above. A specimen without irradiation was used as negative control.

The bactericidal LR for each irradiation dose is given in log_10_ levels and was calculated according to Eq. (1), where *n*_*c*_ is the number of cfu on control specimen without irradiation and *n*_*p*_ is the number of cfu on irradiated test specimen.

1 $$LR = log_{10} (n_{c} ) - log_{10} (n_{p} )$$

### Skin models

OS-Rep-1 RHEs (Henkel AG & Co. KGaA, Düsseldorf, Germany) were used to study cell viability, radical formation and DNA damage immediately, 24 h after irradiation, and after multiple irradiation. Excised human female stomach and breast skin (Fitzpatrick skin types II–III) without any skin diseases originating from plastic surgeries were used for the determination of immediate DNA damage and radical formation. All experimental protocols were approved by the Ethics Committee of the Charité-Universitätsmedizin Berlin (EA1/324/19). Informed written consent was obtained from the seven participants and all procedures complied with the Declaration of Helsinki.

The removed skin reached the laboratory on the day of surgery and had been disinfected with a solution of 2-propanol 70% before starting surgery. In the laboratory it was cleaned with PBS, excess fatty tissue was removed. Then, the skin was stored at 4 °C on moistened paper until use. All investigations were done within 24 h of receipt.

The RHE models were cultivated in six well plates at 37 °C, 5% CO_2_ and 100% humidity in OS Rep-Air–liquid interface medium (OS-Rep-ALI, CM-125, Henkel AG & Co. KGaA, Düsseldorf, Germany). The medium was changed every 2 days. Due to photosensitivity of the medium, the RHEs were placed into PBS during irradiation.

### Cell viability

The influence of UV irradiation on the skin cell metabolism was controlled by an MTT [3-(4,5-dimethylthiazol-2-yl)-2,5-diphenyltetrazolium bromide] assay^28,29^. Therefore, Ø = 4 mm punch biopsies of excised human skin or RHE were investigated directly after irradiation as previously described^64^.

### Analysis of DNA damage

To assess the effect of UV irradiation on skin cells, 6-4PP and CPD were examined immunohistochemically on 1–2 µm paraffin sections as previously described^24^. The sections were incubated with either anti-6-4PP (clone 64M-2, Cosmo Bio) or anti-CPD (clone TDM-2, Cosmo Bio). Alkaline Phosphatase/RED, Rabbit/Mouse (Agilent Technologies) was employed for the detection of 6-4PP^+^ and CPD^+^. Nuclei staining was performed with hematoxylin (Merck Millipore) and slides were coverslipped with Kaiser’s glycerol gelatine (Merck Millipore). Analysis of apoptosis in irradiated cells was performed by cleaved caspase-3 staining (clone 5A1E, Cell Signaling Technologies) on selected sections^31^. Negative controls were performed by omitting the primary antibody. An AxioImager Z1 microscope (Carl Zeiss MicroImaging, Inc.) was used for histologic documentation in a blinded manner.

### Quantitative radical measurements

Quantitative analysis of free radicals induced by UVC irradiation in excised human skin was performed and epidermal skin equivalents were measured by electron paramagnetic resonance (EPR) spectroscopy, using the spin marker PCA (3-(carboxy)-2,2,5,5-tetramethylpyrrolidin-1-oxyl) (Sigma Aldrich, Steinheim, Germany).

Excised human skin was horizontally dermatomed (Aesculap^®^ Acculan 3Ti, Aesculap, Tuttlingen, Germany) to a thickness of 300 µm, the RHE were around 100 µm thick and were not processed further.

Ø = 3 mm skin punch biopsies were incubated from both sides with 21.6 µl of the spin marker PCA [1.5 mM] by using Ø = 6 mm filters (SmartPractice Europe GmbH, Greven, Germany) for 10 min at 37 °C. The EPR measurements were performed on an X-band EPR spectrometer (Bruker Elexsys E500, BioSpin GmbH, Karlsruhe, Germany) at room temperature. A TMHS resonator (E2044500TMHS, Bruker BioSpin GmbH, Karlsruhe, Germany) was used with the following parameter settings: frequency = 9.5 GHz, central magnetic field = 350.5 mT, magnetic field sweep width = 40 mT, modulation frequency = 100 kHz, modulation amplitude = 0.5 mT, attenuation = 22 dB, sweep time = 45 s.

### Statistical Analysis

All antibacterial efficacy tests were conducted at least in triplicate on distinct samples. DNA damage and cell viability measurements were performed on *n* ≥ 3 distinct samples. Radical formation experiments were conducted with *n* = 3 to *n* = 9 RHE models and *n* = 4 human skin donors with 2 to 3 technical replicates each. Either a Kruskal–Wallis test or a one-way analysis of variance (ANOVA) was conducted to compare antibacterial efficacy. Mean value comparisons were performed with the Kruskal–Wallis test for DNA damage data. Bonferroni correction was applied to account for multiple testing in all cases. An ANOVA with Dunnett post hoc tests was conducted for mean value comparison of radical formation and cell viability in RHEs. Mean value comparison of radical formation in excised human skin was performed by using the Student’s t-test for paired samples. The statistical analysis was executed using IBM SPSS^®^ Statistics 27 (IBM, Armonk, NY, USA) affording a significance level of *α* = 0.05. Display of data was performed in OriginPro 2020 (Origin, OriginLab Corporation, Northampton, MA, USA). All data are expressed as mean ± standard error (SEM).

## Supplementary Information

Supplementary Figures.

## Data Availability

Data that support the findings of this study are available from the corresponding author upon reasonable request.
