# Supplementary material for: Application of 233 nm far-UVC LEDs for eradication of MRSA and MSSA and risk assessment on skin models
Source: Sci Rep. 2022 Feb 16;12:2587. doi: 10.1038/s41598-022-06397-z (PMC8850561; doi:10.1038/s41598-022-06397-z)
Supplement: Supplementary file 1 — Supplementary Figures. [file 41598_2022_6397_MOESM1_ESM.docx]

Supporting Information

Application of 233 nm far-UVC LEDs for eradication of MRSA and MSSA and risk assessment on skin models

Paula Zwicker^1,8^, Johannes Schleusener^2,8^, Silke B. Lohan^2^, Loris Busch^2,3^, Claudia Sicher^1^, Sven Einfeldt^4^, Michael Kneissl^4,5^, Anja A. Kühl^6^, Cornelia M. Keck^3^, Christian Witzel^7^, Axel Kramer^1^ and Martina C. Meinke^2^*

^1^ Institute of Hygiene and Environmental Medicine, Ferdinand-Sauerbruch-Str., University Medicine Greifswald, 17475 Greifswald, Germany

^2^ Charité – Universitätsmedizin Berlin, Corporate Member of Freie Universität Berlin and Humboldt-Universität zu Berlin, *Center of Experimental and Applied Cutaneous Physiology, Department of Dermatology, Venerology and Allergology,* Charitéplatz 1, 10117, Berlin, Germany

^3^ Department of Pharmaceutics and Biopharmaceutics, Philipps-Universität Marburg, Robert-Koch-Str. 4, 35032 Marburg, Germany

^4^ Ferdinand-Braun-Institut gGmbH, Leibniz-Institut für Höchstfrequenztechnik, Gustav-Kirchhoff-Str. 4, 12489 Berlin, Germany

^5^ Institute of Solid State Physics, Technische Universität Berlin, Hardenbergstr. 36, 10623 Berlin, Germany

^6^ iPATH.Berlin-Immunopathology for Experimental Models, Core Facility of the Charité – Universitätsmedizin Berlin, Corporate Member of Freie Universität Berlin and Humboldt-Universität zu Berlin, Charitéplatz 1, 10117, Berlin, Germany

^7^ Charité – Universitätsmedizin Berlin, Corporate Member of Freie Universität Berlin and Humboldt-Universität zu Berlin, Division of Plastic and Reconstructive Surgery, Department of Surgery, Charitéplatz 1, 10117, Berlin, Germany

^8^ These authors contributed equally: Paula Zwicker, Johannes Schleusener.

* Corresponding author: Martina C. Meinke: martina.meinke@charite.de


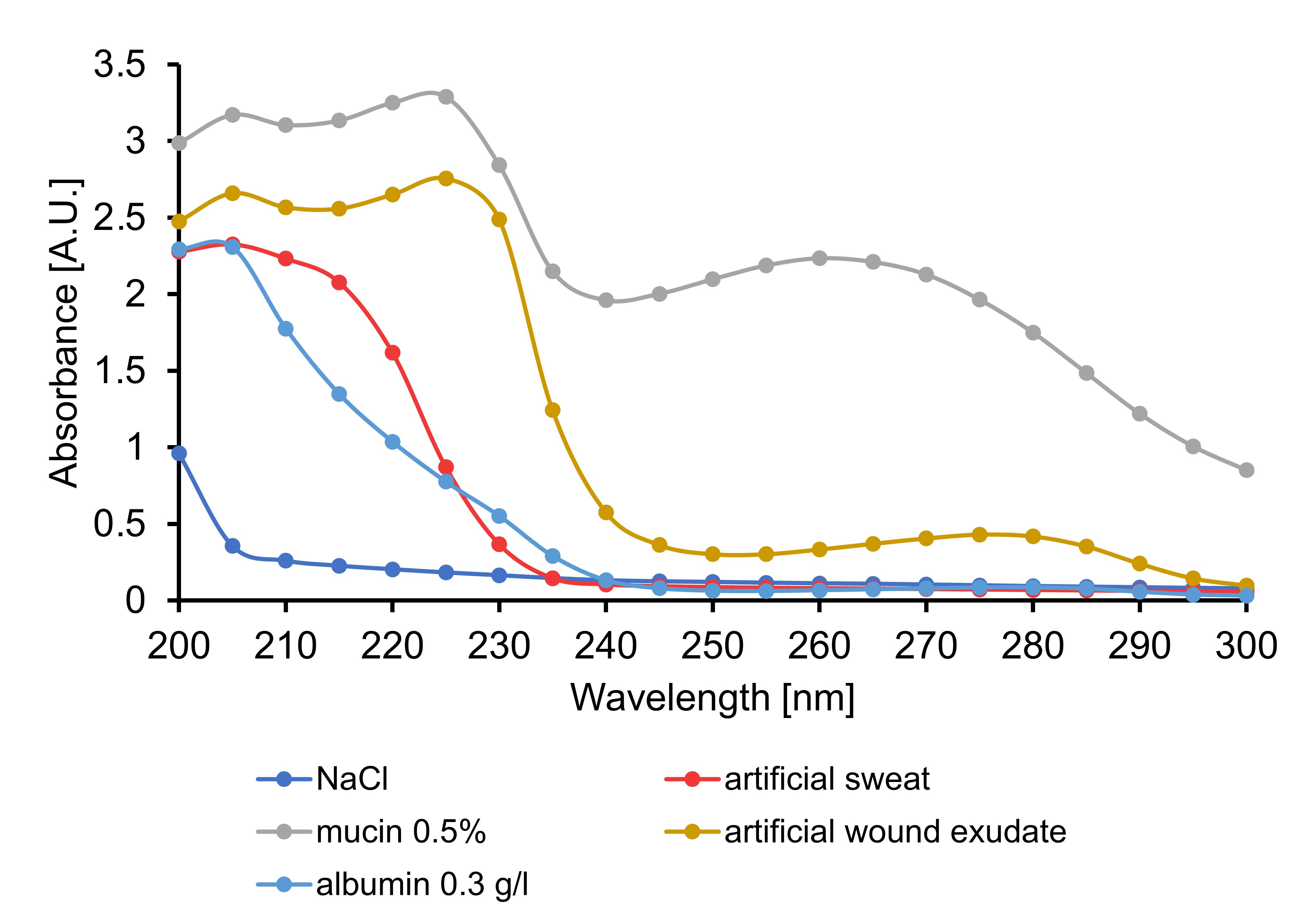


Fig. S1 | Absorption spectra of soil loads. At 230 nm, absorption was highest for mucin and the artificial wound exudate. For 222 nm the absorption was even higher. Sodium chloride solution has the lowest observed absorption at the relevant wavelengths.


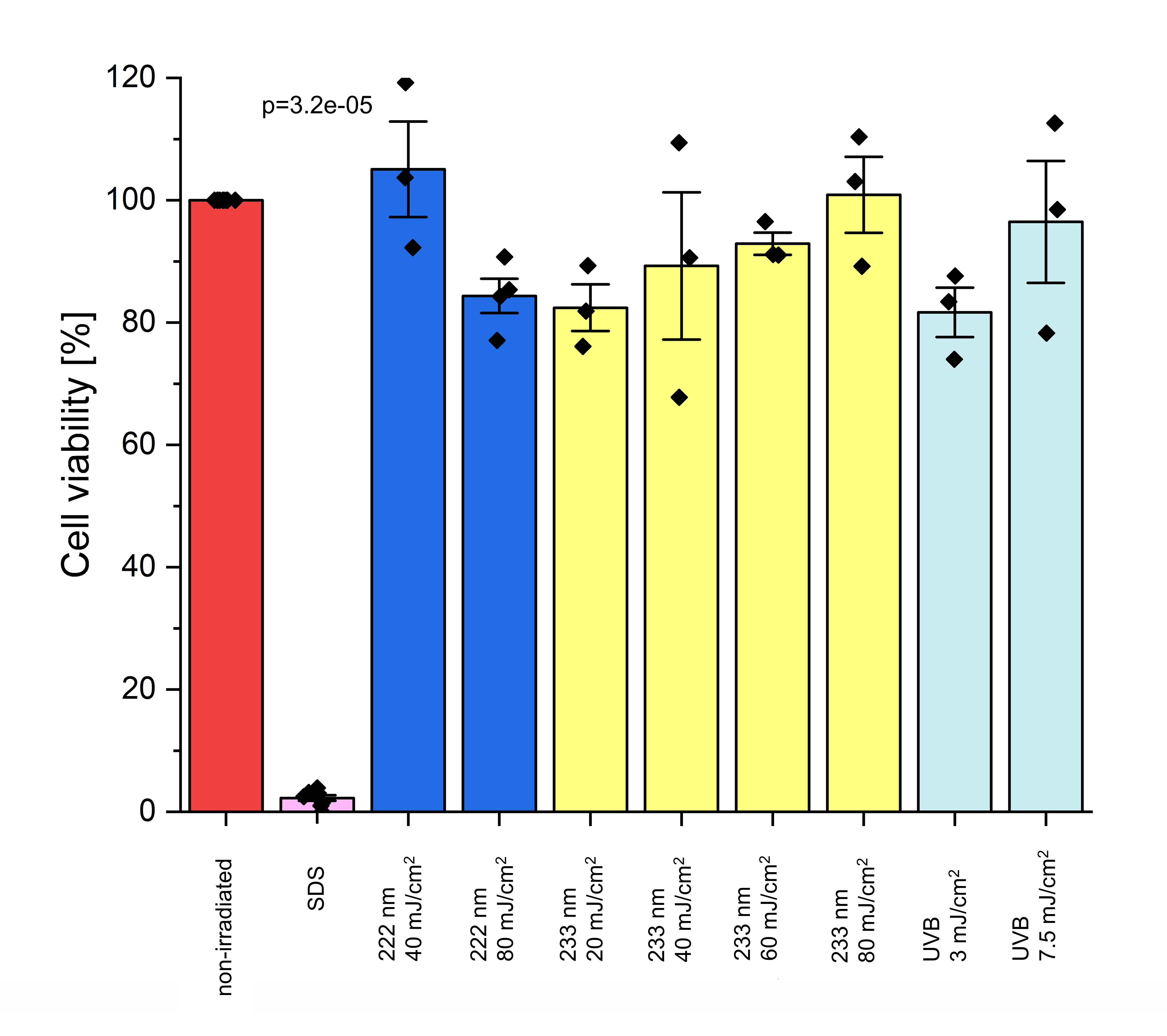


Fig. S2 | Cell viability of RHEs after UV irradiation. The data show the percentage of cell viability after UV irradiation of RHEs in relation to non-irradiated RHEs (negative control, 100% viability, red) determined by an MTT test. As a positive control, RHEs were incubated for 1 h in sodium dodecyl sulphate (SDS, rosé), entailing (2.1±0.5)% cell viability. Reduction below 80% was not observed. The statistical significance was tested by Dunnett’s post hoc tests after one-way ANOVA by testing each group against the negative control. The data show mean±SEM. *n*=3–9.


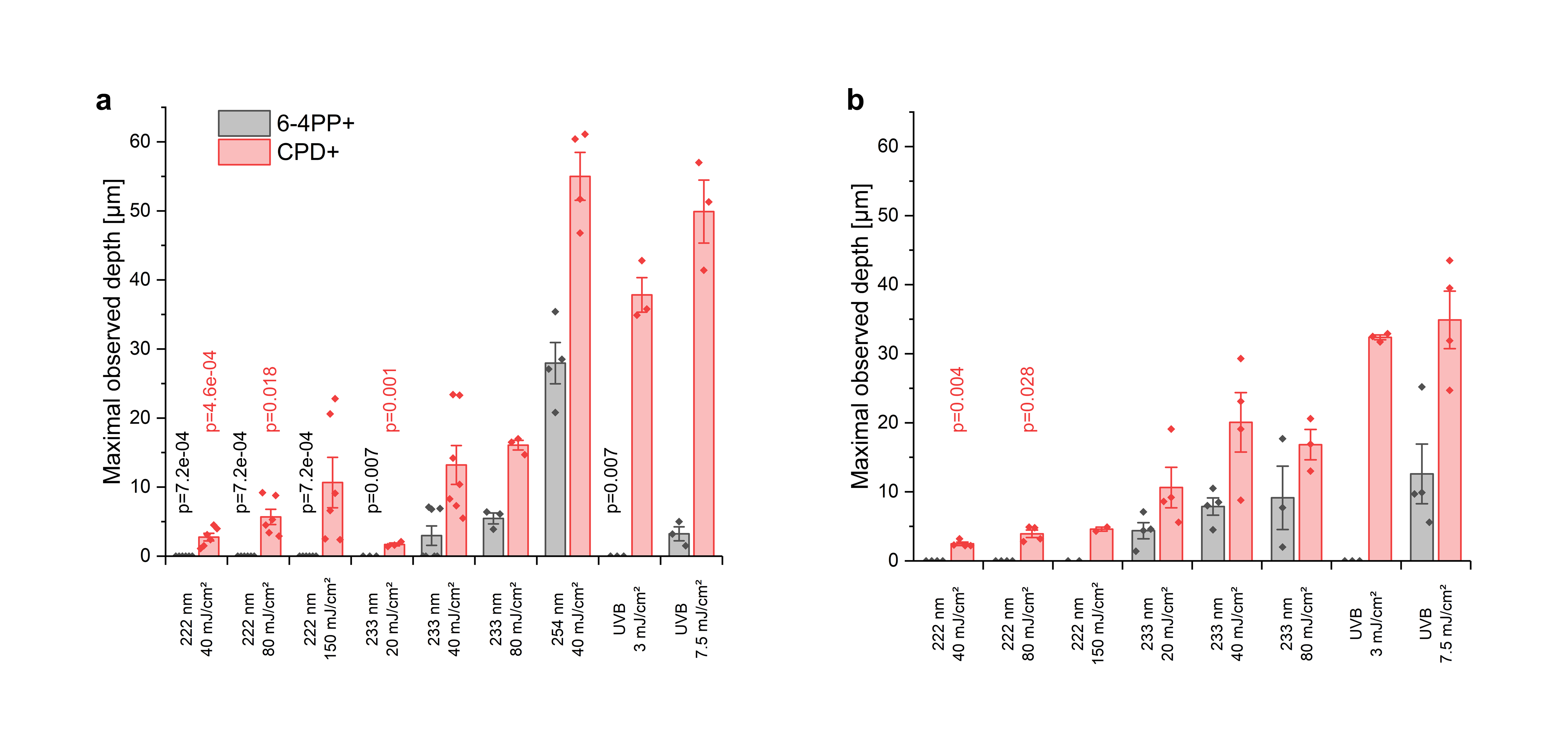


Fig. S3 | Depth of DNA damage in the viable epidermis for RHEs (a) and excised human skin (b). The maximal observed depth is measured from the skin surface to the deepest occurrence of 6–4PP^+^ (grey) and CPD^+^ cells (red) cells on five positions in the histology images; the SC thickness is subtracted for comparability. The p-values are based on Bonferroni-adjusted post hoc tests executed after Kruskal-Wallis ANOVA. For RHEs (a) each group was tested against 254 nm 40 mJ/cm^2^ and for excised human skin (b) each group was tested against UVB 3 mJ/cm^2^. In (b) the maximal observed depth of DNA damage after irradiation with 222 nm at 150 mJ/cm2 is not included in the ANOVA since the sample size was only n=2. The data show mean±SEM. *n*=2–7.
